# Diabetic Kidney Disease: Contribution of Phenyl Sulfate Derived from Dietary Tyrosine upon Gut Microbiota Catabolism

**DOI:** 10.3390/biom14091153

**Published:** 2024-09-13

**Authors:** Haoxin Liu, Tram N. Diep, Ying Wang, Yucheng Wang, Liang-Jun Yan

**Affiliations:** 1 Department of Pharmaceutical Sciences, UNT System College of Pharmacy, University of North Texas Health Science Center, Fort Worth, TX 76107, USA; haoxinliu@my.unthsc.edu (H.L.); tramdiep@my.unthsc.edu (T.N.D.); 2 Institute of Medicinal Biotechnology, Chinese Academy of Medical Science and Peking Union Medical College, Beijing 100050, China; wangying@imb.pumc.edu.cn (Y.W.); wangyucheng@imb.pumc.edu.cn (Y.W.)

**Keywords:** gut microbiota, diabetic kidney disease, tyrosine, phenyl sulfate, oxidative stress, mitochondrial dysfunction

## Abstract

Deranged gut microbiota can release increased levels of uremic toxins leading to exacerbated kidney injury. In diabetic kidney disease (DKD), phenyl sulfate (PS) derived from tyrosine catabolism by gut microbiota has been demonstrated to be both an early diagnostic marker and a therapeutic target. In this perspective article, we summarize PS generation pathways and recent findings on PS and kidney injury in DKD. Increasing evidence has shown that the underlying mechanisms of PS-induced kidney injury mainly involve oxidative stress, redox imbalance, and mitochondrial dysfunction, which all may be targeted to attenuate PS-induced kidney injury. For future research directions, we think that a deeper understanding of the pathogenic role of PS in kidney injury using a variety of diabetic animal models should be investigated. Moreover, we also suggest beneficial approaches that could be used to mitigate the deleterious effect of PS on the kidney. These approaches include caloric restriction, tyrosine restriction, and administration of ketogenic drugs, ketogenic diets or natural products; all of which should be conducted under obese and diabetic conditions.

## 1. Introduction

Diabetic kidney disease (DKD) is a severe complication of both type 1 and type 2 diabetes mellitus [1]. It is a leading cause of end-stage renal disease [2]. Approximately 30% to 40% diabetic patients can develop DKD [3,4]. The hallmark of this chronic kidney disease (CKD) is an elevated level of albuminuria, progressive kidney fibrosis, and a decreased rate of glomerular filtration [5,6,7]. Current managements of DKD include targeting hypertension and hyperglycemia as well as managing dyslipidemia and obesity [8,9,10]. Despite all these approaches that can potentially slow the progression of DKD, no effective treatments for DKD are currently available. Therefore, there is an urgent need to further understand the pathophysiology of DKD and to find effective therapeutic targets or approaches that can be used to combat DKD.

## 2. Gut Microbiota and Kidney Injury and the Usefulness of Animal Models

It has been well established that gut microbiota plays an important role in human health and disease [11,12,13]. While there is evidence through twin studies that certain components in gut microbiota are heritable [14], environmental factors such as diet, drugs, and toxicants can also determine the composition of gut microbiota [15]. Lifestyle factors such as smoking and alcohol drinking can also alter gut microbiome [16,17]. Therefore, gut microbiota has been thought to be a virtual organ that can dictate a given individual’s health and disease [13]. Particularly, when disturbed or disrupted, the gut microbiota can undergo a homeostatic imbalance leading to dysbiosis reflected by a diminished diversity [18,19,20]. Under such conditions, numerous microbiota metabolites tend to accumulate in the body and become uremic toxins, which then overload the kidney and cause kidney injury [21]. This is indeed the case for the amino acid tyrosine that is catabolized by deranged microbiota to produce free and protein bound uremic toxins such as p-cresyl sulfate and phenyl sulfate [18,22,23]. In this perspective article, we would like to focus on phenyl sulfate (PS) and its effects on diabetic kidneys as increasing evidence has established that PS can be used as an early DKD marker and also as a therapeutic target [23,24].

At this point before further discussion, we would like to point out that in the research area of gut microbiota studies, animal models have provided most of the knowledge garnered so far on gut microbiome and gut bacteria [25]. They help elucidate regulatory and metabolic mechanisms and identify intestinal microorganisms. Nonetheless, findings using animal models concerning gut microbiota and its effects on health and disease are yet to be translated to humans [23]. Nevertheless, animal models related to gut microbiota research should and will continue to find their niche in our understanding of the relationship between gut microbiota and kidney health and disease.

## 3. Catabolism of Dietary Tyrosine by Gut Microbiota and Generation of Phenyl Sulfate

**Figure 1:**
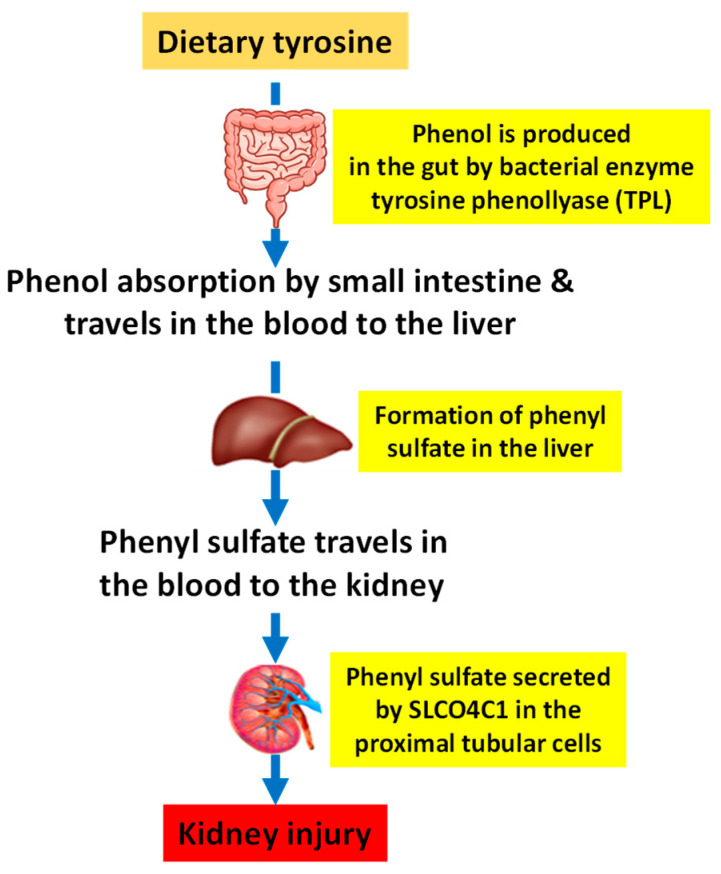
Major pathway of phenyl sulfate formation via gut microbiota catabolism of dietary tyrosine. Tyrosine is converted to phenol by the bacterial enzyme tyrosine phenol lyase followed by further conversion to phenyl sulfate in the liver. Phenyl sulfate is usually eliminated by the kidney but can accumulate in the kidney and cause further kidney damage.

Following gut bacterial catabolism of tyrosine, phenol is produced along with another tyrosine derivative p-cresol [23]. The phenol formation reaction is catalyzed by a bacterial enzyme in the gut known as tyrosine phenol lyase (TPL) and the formed phenol molecule can then be absorbed by the small intestine and travels in the blood to the liver whereby it is converted to phenyl sulfate (PS) [23]. PS is then released from the liver and travels in the blood to the kidney whereby it is secreted by a channel named SLCO4C1 in the proximal tubular cells (Figure 1) [24]. Therefore, a pre-existing kidney disorder can cause PS accumulation in the kidney and exacerbates kidney dysfunction [26], as in DKD.

The seminal study by Kikuchi et al. has shed more light on the role of PS in DKD [24]. They found that PS could serve as a biomarker for early diagnosis of DKD, could be a risk factor for kidney injury and albuminuria, and could be used to predict DKD progression. Therefore, PS could potentially be a therapeutic target for DKD.

## 4. Animal Model of Diabetic Nephropathy Induced by PS

Since the publication of Kikuchi’s paper [24], PS, as an add-on detrimental factor for DKD, has been further shown to worsen DKD by others. Zhang et al. recently confirmed that PS can be used to create a DKD model using db/db mice [27]. The role of PS in such a model is likely to speed up DKD development by high levels of PS in the background of db/db mice. The authors used this PS-induced DKD model as a platform to further test the protective effects of a traditional Chinese medicine called Huajuxiaoji (HJXJ) and found that HJXJ could alleviate PS-induced DKD. HJXJ decreased the levels of blood urea nitrogen, creatinine, urinary protein content, and inflammatory cytokines such as interleukin (IL)-18, IL-1β, IL-6, and tumor necrosis factor α. HJXJ was also found to inhibit NLRP3 in diabetic kidneys. This study further demonstrates that the PS-induced DKD model in the background of pre-existing diabetes can be used as a platform to test the anti-DKD effects of traditional Chinese medicine and other natural antioxidant products as well as artificial therapeutic agents.

It should be noted that whether sole exogenous PS exposure in the absence of pre-existing diabetes can be used to create an animal model of kidney injury mimicking DKD remains to be investigated in the future. If such a model is feasible, it would greatly facilitate research in the area of the gut–kidney axis encompassing PS-induced renal toxicity, the effects of deranged gut microbiota on kidney function, and testing the potential capacity of a given natural product or a drug on PS-induced kidney injury.

## 5. Pathological Mechanisms of PS-Induced Kidney Injury

**Figure 2:**
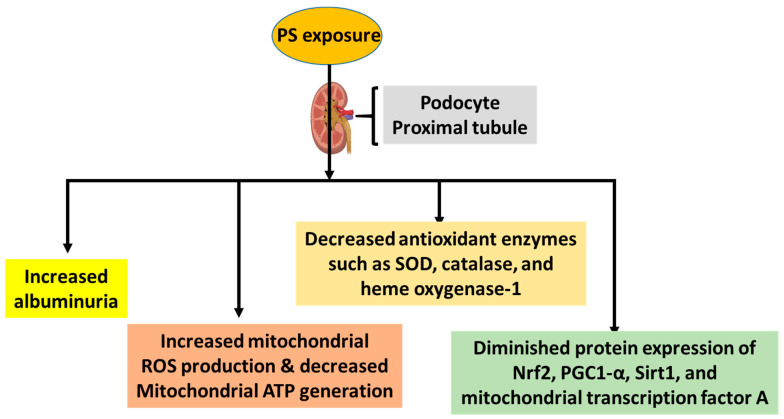
Major mechanisms underlying PS-induced kidney injury.

While it is known that PS content elevates with the progression of diabetes and such an elevation correlates with increased albuminuria via podocyte injury [24,28], any potential deleterious effects on tubular cells in vivo have not yet been fully assessed, although cell culture studies on the deleterious effects of PS on renal tubular cells have been reported [29,30]. With respect to podocyte injury, the underlying mechanisms of PS have been shown to involve increased mitochondrial generation of reactive oxygen species and mitochondrial dysfunction concurrent with decreased mitochondrial ATP production [24]. Additionally, levels of glutathione and antioxidant enzymes including catalase, superoxide dismutase, and heme oxygenase-1 were also decreased by PS exposure [28,29]. All these impairments also show correlation with diminished protein expression of Nrf1, PGC1-α, Sirt1, and mitochondrial transcription factor A [28] (Figure 2). It should also be noted that gut and renal inflammation is also implicated in PS-induced kidney dysfunction [31,32]. As a result, all these mechanisms may be targeted to attenuate PS-induced kidney injury.

## 6. Potential Approaches for Mitigating PS-Induced Kidney Injury

In healthy individuals, PS (both free and protein bound) may be effectively eliminated by healthy kidneys. On the contrary, a dysfunctional kidney, such as in the situation of chronic kidney disease (CKD) and DKD, can cause PS accumulation in the blood and within the kidney [26,33], accentuating the injury of a kidney whose function is already impaired. This is likely the reason for the use of PS to induce kidney injury in pre-existing diabetic animals along with the fact that pre-existing diabetes drives the elevation of microbiota-derived PS [24,27,28]. Given that PS can contribute to kidney injury in diabetes, several strategies may be considered for counteracting PS-accentuated DKD. One strategy would be to inhibit or block the bacterial enzyme tyrosine phenol lyase (TPL) [24,34]; another would be to restrict dietary tyrosine intake [35,36]. Nonetheless, a comprehensive evaluation of dietary tyrosine restriction is yet to be conducted. Table 1 gives some of the strategies that can be considered for attenuating PS effects on kidney injury.

Additionally, in DKD as well as in CKD, the microbiota environment and microbiota structure are deranged, leading to the production of elevated levels of PS that is responsible for further renal damage [42,43]. Therefore, oral intake of prebiotics, probiotics, and dietary antioxidant supplements may also serve as approaches for ameliorating dysbiosis and combating PS-accentuated DKD [44].

## 7. Future Directions

The deleterious effects of PS on diabetic kidneys have mainly come from a handful of studies using db/db mice, streptozotocin (STZ)-induced diabetic animals, Akita mice, and KK-Ay diabetic mice [45] treated with a high-fat diet (HFD) [24,27]. We think that research should be expanded to encompass other widely used animal models of diabetes such as HFD-STZ diabetic models [46,47,48]. Moreover, dietary manipulation strategies such as caloric restriction [49,50], methionine restriction [51,52], and ketogenic drugs or ketogenic diets [53,54,55,56] should also be comprehensively evaluated for PS effects on kidney dysfunction. Table 2 lists widely used animal models and dietary manipulation strategies that await future investigations. Table 2 also lists some of the widely used CKD models such as those induced by adenine [57] and folic acid [58]. Mechanistically, future studies should also explore PS effects on the endoplasmic reticulum (ER) and the crosstalk between ER and mitochondria [59,60]. Mitochondrial Sirt3 function and NAD^+^-dependent redox signaling and redox imbalance [61,62] upon PS exposure in diabetic kidneys also remain to be studied. A complete profiling of PS revolving around the gut microbiota and kidney axis in obesity conditions without persistent hyperglycemia should also be investigated. It should be noted that as a potential biomarker of DKD, at what early DKD stage could we detect increased PS should also be comprehensively evaluated.

## 8. Summary

The gut microbiota and kidney axis is intricately involved in a bi-directional relationship between microbiota metabolism and kidney health and disease [72,73,74]. In diabetes, there is an increased level of the uremic toxin PS derived from dietary tyrosine upon gut microbiota catabolism of tyrosine. PS can accentuate DKD and the underlying mechanisms likely involve oxidative stress, mitochondrial dysfunction, and compromised cellular antioxidant capacities. PS is both an early DKD diagnostic biomarker and a therapeutic target [23,75]. To further elucidate the mechanisms of PS-induced kidney injury, other animal models of diabetes should be evaluated along with CKD animal models (Table 2). Moreover, the effects of dietary manipulations such as caloric restriction and ketogenic drugs or ketogenic diet on microbiota’s PS production in the context of diabetes should also be investigated. All these further studies may unravel more therapeutic targets along the PS metabolic and signaling pathways.

## Figures and Tables

**Table 1:** Strategies for attenuating the deleterious effects of PS on the kidney.

1. Tyrosine phenol lyase inhibitors or blockers [24,34];
2. Decrease in dietary tyrosine intake or dietary tyrosine restriction [28,29];
3. Adsorption of protein-bound PS through direct hemoperfusion [37,38];
4. Natural products possessing anti-oxidation and anti-inflammation properties [27,39,40,41].

**Table 2:** Animal models and dietary manipulation strategies to be evaluated.

1. High-fat diet/Streptozotocin [46,47,48]
2. High fructose/Streptozotocin [63,64]
3. Nicotinamide/Streptozotocin [65]
4. Zucker diabetic rats and ZSF-1 rats [66,67,68]
5. Caloric restriction/energy restriction [49,50,69]
6. Dietary methionine restriction [51,52]
7. Ketogenic drug or ketogenic diet [53,54,55,56]
8. Adenine-induced chronic kidney disease [31,57]
9. High folic acid-induced chronic kidney disease [58]
10. Metal-induced kidney injury [70,71]
